# Magnitude and associated factors of occupational hazard exposures among sanitary workers: Propose RASM model for risk mitigation for the public hospitals, eastern Ethiopia

**DOI:** 10.1186/s13104-024-06828-2

**Published:** 2024-06-20

**Authors:** Sina Temesgen Tolera, Nega Assefa, Abraham Geremew, Elka Toseva, Tesfaye Gobena

**Affiliations:** 1 College of Health and Medical Sciences, Haramaya University, P.O. Box: 235, Harar, Ethiopia; 2 Department of Hygiene, Faculty of Public Health, Medical University of Plovdiv, Plovdiv, Bulgaria

**Keywords:** Associated factors, Hazards, Magnitude, Occupational health, Risk mitigation, Sanitary workers

## Abstract

**Background:**

Hospital sanitation workers (SWs) are exposed to numerous occupational hazards due to workplace conditions such as unsafe and unhygienic working environment in the hospitals. Therefore, knowing magnitude, types and source of occupational hazard exposures with their determinants are very significant for further mitigations.

**Methods:**

Hospital based cross-sectional study design was conducted in public hospitals, eastern Ethiopia from 1st May to August 30th, 2023. 809 SWs participated. Data was entered into Epi Data Version 3.1 and Stata 17MP version used for analysis. Descriptive analysis was applied to describe the data. While, multilevel logistic regression was explored to determine the association between outcome and independents among at individual level (model 1), at hospitals (model 2) and combination of the two (model 3). The crude odds ratio (COR) and adjusted odds ratio (AOR) for models 2 and 3 were reported. Variables with an AOR with a 95% confidence interval (CI) at a p-value < 0.05 were reported.

**Result:**

Out of 809 SWs, 729 (90.11%) responded. The overall magnitude of self-reported occupational hazard exposures among SWs was 63.65% (95% CI 0.60–0.67). Of this, biological, chemical, and ergonomic hazards accounted for 82.44%, 74.76%, and 70.92%, respectively. The multilevel logistic regression shows that having social recognition (AOR: 0.37, 95% CI 0.14, 0.91), neutral attitude (AOR: 0.48, 95% CI 0.17, 1.41) as compared to negative attitude. The model also found that SWs those supervised could reduce the likelihood of occupational hazard exposures by 50% times (AOR: 0.50, 95% CI 0.18, 1.38) as compared to non-supervised SWs. The final model predicted the variation of occupational hazard exposures among sanitary workers from the hospitals to hospitals was 26.59%.

**Conclusions:**

The concluded that hospital sanitary workers are facing biological, chemical, ergonomic, physical, psychological, mechanical, and electrical hazards. This study’s findings predicted that dissatisfied with their environment, working more than 8 hr per a day,  a negative attitude towards workplace risks and inadequate supervision may serve as contributing factors for the likelihood of occupational hazard exposures among these groups. Thus, the study suggested that hospitals could reduce these hazard risks if they implement the Risk Assessment and Safety Management (RASM) model, which includes multi-modal strategies, indicators and tripartite philosophy.

**Supplementary Information:**

The online version contains supplementary material available at 10.1186/s13104-024-06828-2.

## Introduction

Occupational hazards are defined as the potential source of injury or poor health effect on a person or individuals arising from any unsafe working environment due to insufficient occupational health and safety (OHS) implementation [1]*.* Occupational health is a branch of public health that works to promote and maintain the best level of physical, mental, and social well-being among workers in all jobs [2]. On the other hands, Occupational safety refers to the goal of reducing the risk of dangers that may arise as a result of events connected to the tasks that workers undertake in the workplaces, particularly when equipment are utilized [2].

Now-a-day assessing occupational hazard exposures (OHE) is crucial because currently result in a growing amount of financial loss as well as intangible damage within businesses globally [3]. Despite it is not well identified among SWs, those are providing a service for large community across the world by cleaning health facilities and other work setup [4–6]. They frequently cited aspects of working circumstances that are common not just in low-income countries, such as hazardous working environments, machine safety, unsanitary workplaces, high temperatures, excessive noise, and poor indoor air quality [7]. Moreover, they are exposed to chemical, biological, ergonomically, mechanical, electrical, psychological hazards [8, 9]. Despite of fact that different workplaces may have hazards and their frequency varied according to workplace or settings—with their attitude levels and practice levels [10].

According to the findings of an Egyptian research, the most prevalent dangers among these categories were psycho-social (76.60%) and biological hazards (65.60%) [11]. The same study conducted in Thailand showed that the highest prevalence of OHE among was ergonomic (89.3%), and followed by psychological (80%) [12]. The other study found from Nigeria indicated that the highest prevalence among these groups was chemical (77.5%) and followed by physical (55.8%) [13]. The study found from China revealed that the highest exposure rate was psycho-social (85.93%), followed by accidental (70.78%) [14]. The study conducted in Texas, USA showed that slip, fall, hit, caught, waste handling were 85% for contusion, 31% for puncture and cut [15].

The impact of poor health and safety is not only for sanitary workers concerns, but also for damaged goods, institution reputation, legal issues, increased cost and turnover; and decreased service and productivity [16]. The findings indicate that institutional concerns have an impact on sanitary workers’ health as a result of inadequate law enforcement, a lack of legal protection, a lack of standard procedures, and poor work design or pattern [17, 18]. Some of developing countries have guidelines and laws do exist, but governments may lack the financial or technical means to implement [6, 19]; lack of personal protective equipment (PPE).

At national level, Ethiopia, a number of shortcomings in OHS implementation were research gaps, training gaps, capacity gaps, policy and regulatory gaps, organizational gaps, and monitoring and evaluation gaps [20]. In addition, only few studies conducted on magnitude of OHE such as physical, chemical, biological, electrical, mechanical; and psycho social hazard exposures and non mitigation for the workers including SWs in health care facilities at national level.

**Fig. 1:**
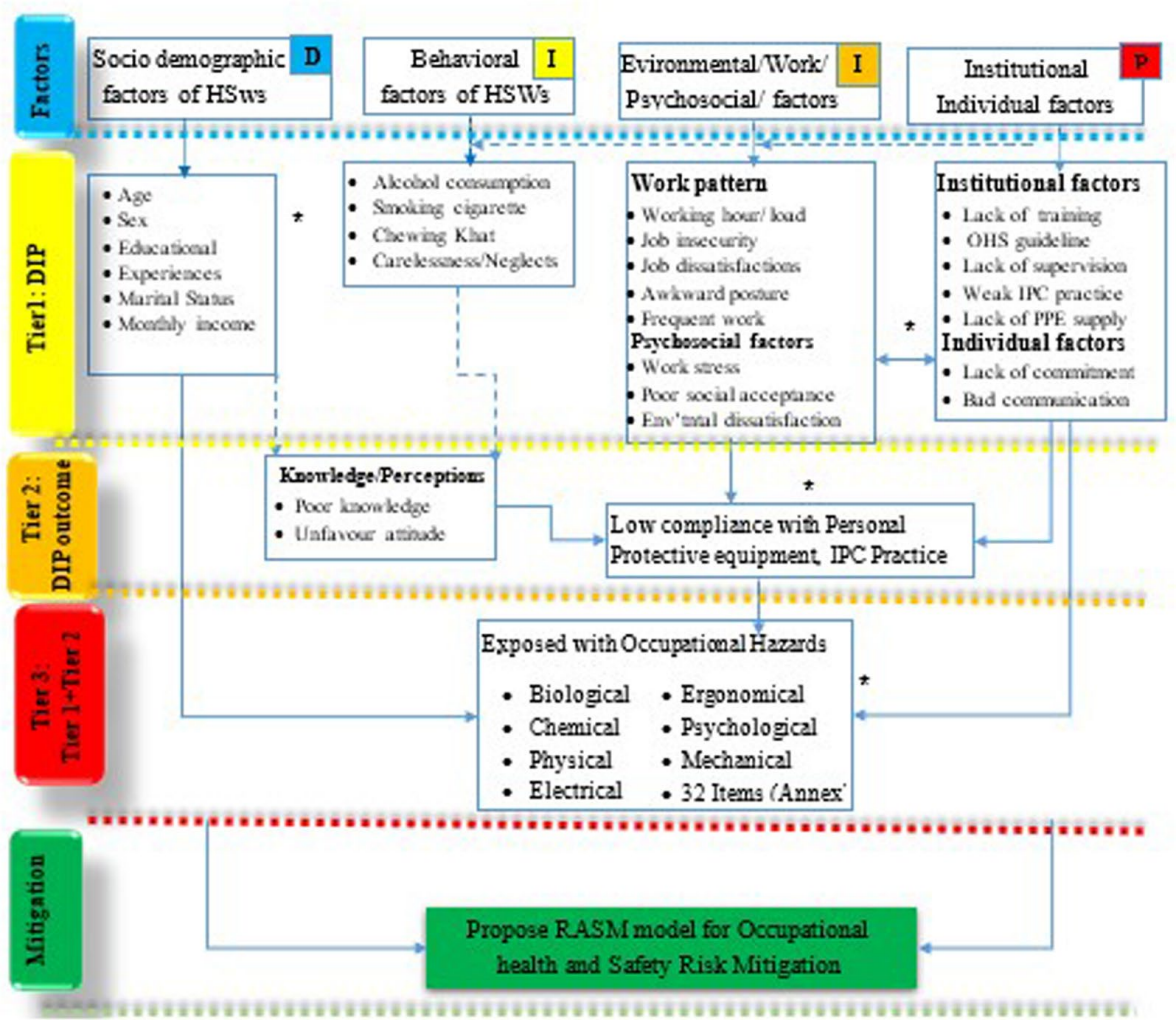
Magntitude and aasociated factors of occupational hazard exposures among sanitary workers in public hospitals, eastern Ethiopia: propose RASM Model for risk mitigation. In Figure, un-break shows “arrows” direct factors (possibilities); break “arrows” indirect factors (probabilities]; asterisk [*] indicating identified hazards used for RASM model

Therefore, the current study aim to assess magnitude and determinants of occupational hazard exposures, adapted from [21] and propose RASM Model for risk mitigation from SWs in public hospitals, eastern Ethiopia, which was adapted from Curtis [22], including interventions [23, 24]. To achieve this goal, the fundamental conceptual aim of the research, which is based on examined evidence, is as follows (Fig. 1).

## Methods

### Study settings and period

**Fig. 2:**
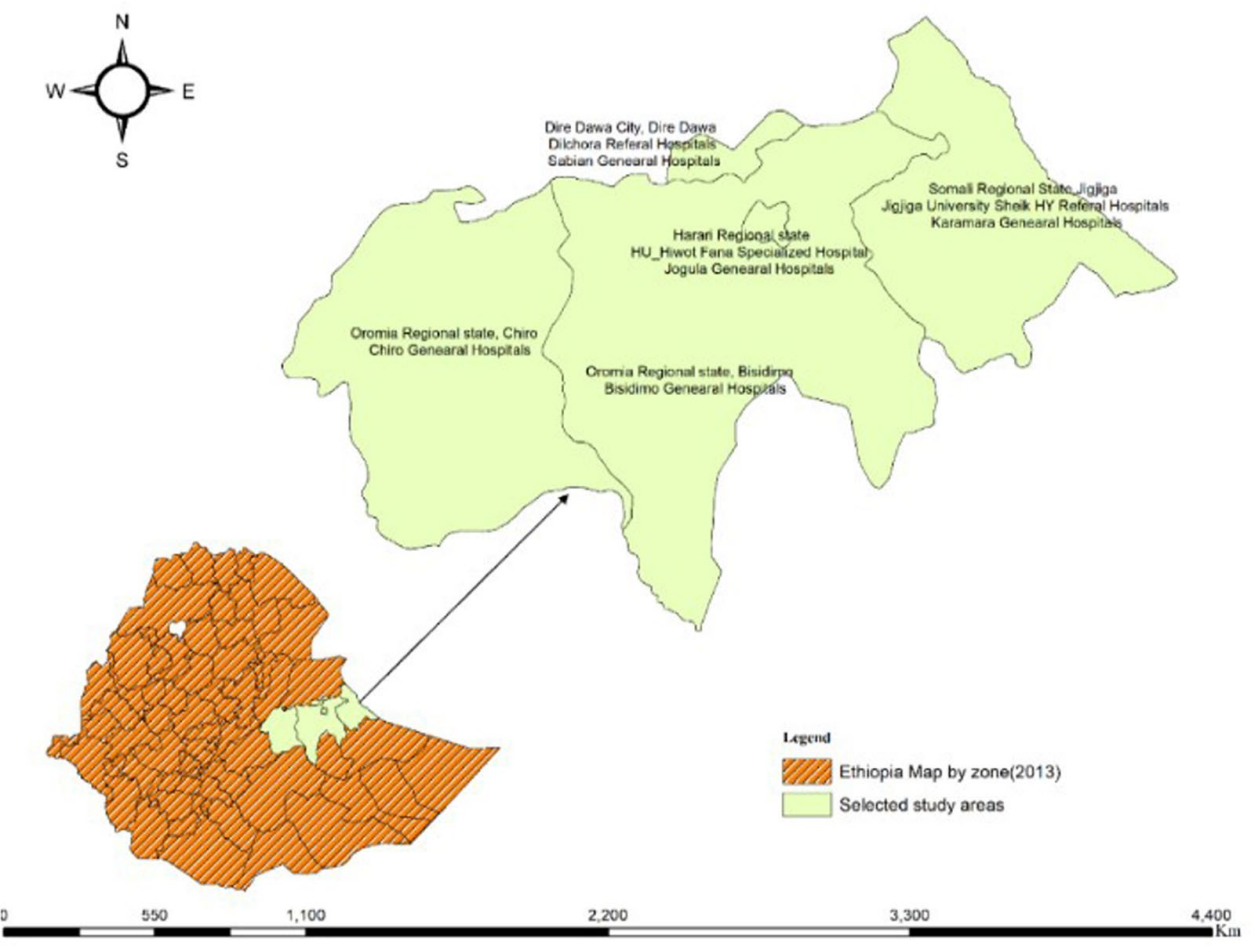
Map of Ethiopia, selected eastern Ethiopia and selected public hospitals for the study created using ARCGIS from free access of Ethiopia GIS datasets

The study was conducted in eastern Ethiopia on eight selected public hospitals (Fig. 2), from 1st May to August 30th, 2023. Eight of them randomly selected from 14 hospitals by providing two hospitals for each of regional state in the eastern Ethiopia.In the daily bases (mean ± SD), the number of bed occupancy in these eight selected hospitals were 269.5 ± 132.6. While, the hospitals providing about 388.3 ± 190.8 for both outpatients and inpatients per a day. Of these, 117.50 ± 57.30 and 198.30 ± 118.20 them were outpatient and inpatient, respectively.

### Study methods

**Fig. 3:**
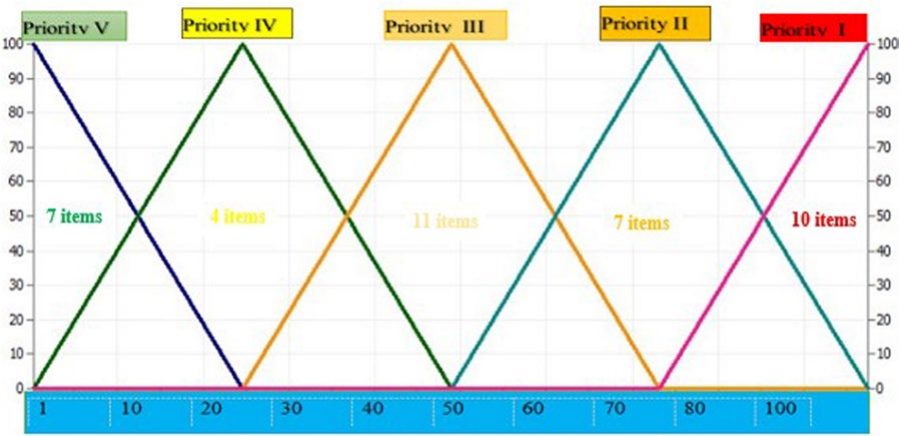
Fuzzy risk index values for potentially identified occupational hazards in hospitals, 2023

Study methods included study design, study populations, inclusion and exclusion criteria, sample determination, selection procedures (Fig. 3), study variables, data collection tools, data quality and data analysis (Supplementary one).

## Result

### Sociodemographic characteristics

Out of 809 SWs, 729 (90.11%) were responded. The majority of them were female (98.49%), 24–35 years old (48.01%), married (69.41%), cleaners (93.14%), permanents (97.26%) and shift one (49.38%). The mean ± SD for age, work experience, educational status, and monthly income salary were 34.35 ± 7.60, 6.65 ± 6.36, 6.78 ± 2.51, and 36.32 ± 6.68, respectively (Table 1).

**Table 1:** Sociodemographic status of SWs in selected public hospitals in eastern Ethiopia, 2023

Demographics	Classification	Frequency (No)	Percentage (%)	Mean ± SD
Sex	Female	718	98.49	
	Male	11	1.51	
Age	≤ 24 years	63	8.64	
	25–35 years	350	48.01	34.35 ± 7.60
	> 35 years	316	43.35	
Work experience	≤ 2 years	133	18.24	
	3–5 years	288	39.51	6.65 ± 6.36
	> 5 years	308	42.25	
Educational status	≤ Grade 4th	160	22.07	
	Grade 5–8th	283	39.03	6.78 ± 2.51
	> Grade 8th	282	38.90	
Marital status	Single	142	19.48	
	Married	506	69.41	
	Separated	59	8.09	
	Divorced	22	3.02	
Income monthly salary (USD [$]	≤ $20.15USD*	12	1.65	
	$20.16–42.95**	672	92.18	36.32 ± 6.68
	> $42.95USD	45	6.17	
Job categories	Cleaners	679	93.14	
	Waste collectors	50	6.86	
Employment type	Permanents	709	97.26	
	Contracts and other	20	2.74	
Type of shift during study	Shift 1	360	49.38	
	Shift 2	262	35.94	
	Shift 3	106	14.54	

Level I* (20.15 = 1100ETB); Level V** (2344ETB), salary classification based on national Job Evaluation and Grading [JEG], 2019] (where 1Dollar ($) = 54.58 ETB, 2 June, 2023

### Self-reported of occupational hazards exposure

The overall burden of self-reported occupational hazards exposure among SWs during period of survey was 63.65% (95% CI 0.60–0.67). From these, the three leading hazards were biological (82.44%), chemical (74.76%) and ergonomics hazards (70.92%) (Table 2).

**Table 2:** Types of self-reported occupational hazards by sanitary workers in public hospitals, 2023

Identified occupational hazards	Correlations and scale test						Responses		Cases
	Obs.	Sign	Item-test	Item-rest	Average interitem	α value	Freq.	%	%
Biological	729	+	0.74	0.62	0.37	0.78	601	18.50	82.44
Chemical	729	+	0.71	0.58	0.38	0.79	545	16.78	74.76
Ergonomics	729	+	0.74	0.61	0.38	0.78	517	15.92	70.92
Physical	729	+	0.65	0.50	0.40	0.80	461	14.19	63.24
Psychological	729	+	0.70	0.57	0.39	0.79	433	13.33	59.40
Mechanical	729	+	0.58	0.42	0.43	0.82	392	12.07	53.77
Electrical	729	+	0.72	0.59	0.38	0.79	299	9.21	41.02
					0.39	0.82		Ave	63.65

The overall correlation of occupational hazard item-test, item-rest and the average inter-item correlation of occupational hazards were presented according to Piedmont et al. [25]. Accordingly, the correlation and scale test show that with respect to this, the average inter-item correlation for a set of items should be between 0.20 and 0.40, suggesting that while the items are reasonably homogenous, they do contain sufficiently unique variance so as to not be isomorphic with each other (Table 2).

### Associated factors of occupational hazards

The final model, multilevel multivariate logistic regression shows that SWs those dissatisfied with their environment (AOR: 5.71, 95% CI 0.70, 46.39) were more increase the likelihood of occupational exposures. However, those worked less than 8 h/day (AOR: 0.50; 95% CI 0.34, 4.0], hadn’t bad social recognition (AOR: 0.37, 95% CI 0.14, 0.91), those had neutral attitude (AOR: 0.48, 95% CI 0.17, 1.41) and for those had sometime supervision (AOR: 0.50, 95% CI 0.18, 1.38) were likely to reduce the severity of occupational hazard exposures as compared to those hadn't supervision (Table 3).

**Table 3:** Multilevel multivariate logistic regression model for predictors of self-reported occupational hazard exposures among SWs in public hospitals, eastern Ethiopia, 2023

Categories of variables		Occupational hazard exposures (N = 729)		COR (95% CI)	AOR (95% CI)	
		Yes: (n = 464)	No: (n = 265)			
Attitudinal towards workplace risk	Unfavored	201 (43.32)	110 (41.51)	1	1	
	Neutral	18 (3.88)	10 (3.77)	0.68 [0.47, 0.99]**	0.48 [0.17, 1.41]*	
	Favored	245 (52.80)	145 (54.72)	0.99 [0.69, 1.43]	0.47 [0.14, 1.65]	
Existence of working > 8 h/day	Yes	138 (76.67)	42 (23.33)	1	1	
	No	327 (59.56)	222 (40.44)	0.46 [0.32, 0.68]**	1.17 [0.34, 4.0]	
Existence of work load	Yes	136 (81.44)	31 (18.56)	1	1	
	No	329 (58.54)	233 (41.46)	0.32 [0.21, 0.49]**	1.38 [0.25, 7.56]	
Conducive environmental	Yes	413 (61.98)	254 (38.02)	1	1	
	No	51 (83.61)	10 (16.39)	3.15 [1.57, 6.31]**	5.71 [0.70, 46.39]*	
Social recognition	Yes	276 (66.67)	138 (33.33)	1		
	No	189 (60.00)	126 (40.00)	0.76 [0.56, 1.03]**	0.37 [0.14, 0.91]*	
Unsafe workplace	Yes	59 (76.62)	18 (23.38)	1	1	
	No	401 (61.88)	247 (38.12)	0.50 [0.29, 0.86]**	0.21 [0.04, 1.14] *	
Conducted supervision	No	140 (68.29)	65 (31.71)	1	1	
	Sometimes	136 (59.39)	93 (40.61)	0.68 [0.46, 1.00]**	0.50 [0.18, 1.38]*	
	Daily	188 (63.73)	107 (36.27)	0.82 [0.56, 1.19]	0.34 [0.54, 1.00]	

Model summary	ICC (%)	AIC	BIC	LR	Sensitivity (%)	Specificity (%)
Model 1	22.22	504.08	551.33	0.58	94.02	7.59
Model 2	16.01	888.16	934.02	0.07	84.78	33.96
Model 3	26.59	501.24	572.07	0.61	84.55	28.97

** stands for p-value is statisitically signifcant at <0.2; while * stand for p-vlaue is statisitically significant <0.05

### Acquired occupational diseases

Due to different occupational occupational hazard exposures the acquired occupational related diseases identified among SW in the public hospitals were reported. The self-report obtained from SWs found that asthma and respiratory tract problems was accounted 22.22 percent. In addition, they acquired allergy (8.89 percent), infections (13.33 percent), both bone fracture and dislocation (4.44 percent), kidney problems (37.78 percent) as well as dermatology problems (8.89 percent) (Table 4).

**Table 4:** Self-reported occupational related diseases acquired among sanitary workers in selected public hospitals, eastern Ethiopia, 2023

Type of disease acquired in hospitals	Frequency	Percentage	Cumulative
Allergy	4	8.89	8.89
Allergic, respiratory tract problems	2	4.44	4.44
Asthma, RT problems	10	22.22	22.22
Infections	6	13.33	13.33
Bone fracture and dislocation	2	4.44	4.44
Kidney problems	17	37.78	37.78
Dermatology/skin infection	4	8.89	8.89
Total	45	100.00	100.00

### Occupational hazards by expert evaluation

The experts’ backgrounds and sources of OHE identified by these experts are attached as supplemental two (Sup.Two), which included Sup.Table 1–Table-3. Accordingly, a large percentage of identified OHE throughout eight institutions were classified as having the potential to generate significant risks (Sup.two: Table 1). The minimum educational background is Bachelor Degree and and maximum is MSc/MPH. Their work experience 3 years to 16 years (Sup. Two: Table 2). They identified, the main sources of OHE was hospital work environment (50%) (Sup. Two: Table 3).

### Fuzzy type of risk index

**Fig. 4:**
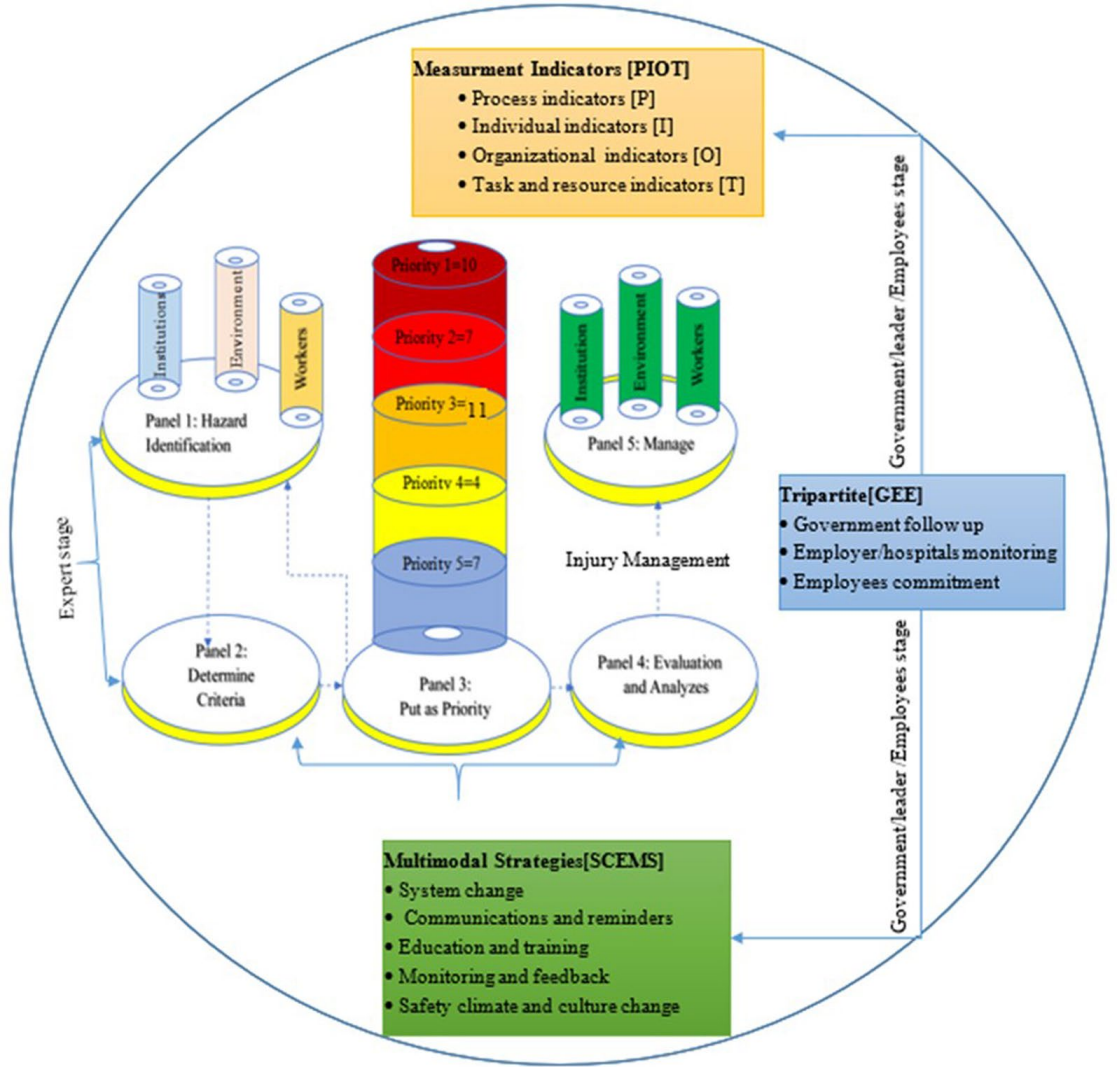
The current overall bridged RASM model was adapted from Curtis [22], Kinney Methods [26], ILO [27] and WHO [28] for risk mitigation in public hospitals, eastern Ethiopia, 2023

Based on systematic evaluation from both infection prevention and control experts as well as sanitary workers, a total 39 items or types of occupational hazard exposure identified. Of these, 32 and 7 of them were identified by experts and sanitary workers, respectively. Of these, 10 items or type of occupational hzards were found at level 1, which the possible potential risk to the sanitary workers, which need first priority for the mitigation. The rest 7 items, 11 items, 4 items and 7 items were reported as level 2, level 3, level 4 and level 5, respectively, which are were moderate risks and tolerance risks, respectively (Fig. 4).

### RASM model for risk mitigation and injuiry management

Based on the probability occupational hazards exposure priority, RASM model could be presented as Glass on Plate considering three routes or dimension. These is because occupational hazard could be one of either instiutution/hospital or/and environment or/and individual problems. In Fig. 4 proposed RASM model is adapted from different philosophical perspective of risk mitigation. The RASM model might be implemented with the help of highly skilled professionals, devoted leaders, employees, and government oversight. Thus, RASM model could be below considering measurement indicators and multi-modal strategies and tripartite scenario. In addition, developing reporting system for occupational impairments including occupational injuries also one of the RASM model activities. RASM model could be achieved well qualified experts as well as well committed leaders, workers including government follow up (Fig. 4).

## Discussion

The overall magnitude of self-reported occupational hazards exposure (63.65%) among sanitary workers (Table 2). As compared to other studies, it was less than 70.2% from Thailand [12]. The disparity may arise from distinct socio-demographic attributes and disparities in risk interpretation knowledge; the current study was conducted in countries with limited development, whereas the previous study was conducted in countries with advanced economies. However, it was higher than 55.05% obtained from Egypt [11]. The discrepancy could be the result of the researchers’ methodological evaluation, since previous study employed a single report, while the present one used cumulative findings.

The current study found that sanitary workers are exposed with multiple occupational hazards. Of these, biological, chemical and ergonomics hazards exposure among hospitals sanitary workers during period of survey were highly prevalence as compared to the other (Table 2). The magnitude of biological hazards was 82.44%, which was higher than 49.40% obtained from Egypt [11], 58.7% obtained from Thailand) [12], 48.6% obtained from Nigeria [13], 63.96% obtained from China [14]. However, the current magnitude of chemical exposure was 74.76% for chemical hazards lower than 77.5% found from Nigeria [13] and 76% obtained from Thailand [12], but higher than 28.60% obtained from Egypt [11], 51.90% found from China [14]. They reported that they exposed ergonomics hazards with 70.92% prevalence, however it was lower than 89.3% obtained from Thailand [12]. In addition, they were exposed to physical hazards at a rate of 63.24%, which was greater than the 55.8% reported in Nigeria [13] and 57.74%) [14]. In addition, the current magnitude of psychological hazards exposure among these was 59.40%, which lower than 80% obtained from Thailand [12], 85.93% found from China [14] and 76.60% obtained from Egypt [11]. As the result of these multiple occupational hazards sanitary workers are acquired occupational diseases. The most self-reported occupational diseases among these groups was asthma and respiratory tract problems was accounted. In addition, they are also acquired allergy, infections, bone fracture and dislocation, kidney problems as well as dermatology problems (Table 4).

The final model, multilevel multivariate logistic regression analysis was performed to determine the occupational hazard exposures and independent variables. Accordingly, model 3 demonstrated that SW who were dissatisfied with the work environment were 5.71 times more likely to be exposed to occupational hazards than those who were happy or satisfied with the work environment (Table 3). Moreover, the study also found that SWs those have social recognition in hospitals were 63% more likely to reduce occupational hazard exposure than those without social recognition. Moreover, SW with no history of sickness or diseases were more likely to lower the risk of occupational hazards by 79% times as compared to those had history of disease. The random effect model revealed found that there was a variance of occupational hazard exposures among SWs from the hospitals to hospitals was 26.59 percent (Table 3). Consequently, the difference in the results was seen, and it was determined that the hospital and/or individual-specific factors could be the cause of this variation in the results from hospitals to hospitals. But 73.41 percent of the variance in work-related occupational hazard exposures was observed among SWs who employed in the same hospitals and this variation might be individual variability.

Along with the evaluation mentioned above, 39 different types of occupational hazard exposures were also assessed by specialists in infection control and prevention in addition to sanitary personnel. Of them, sanitation personnel identified seven, while experts identified thirty-two. Ten (26%) of these items or categories of occupational hazards were discovered to be at the first level. These included working longer than four hours, not managing medical waste well, not receiving enough help from IPC members, and being more likely to be exposed to dangerous chemicals, solvents, and detergents. Concerns about HIV/AIDS, Hepatitis B, and other infections, as well as a lack of personal protective equipment, were raised under this issue. These are the various risks that sanitary personnel may face, and their mitigation must come first. In same manner, moderate occupational risks accounted for 18% of the identified items, making them the second class of risk found. Among these are falls, slides, caught equipment and materials, improper medical waste management transportation, and insufficient awareness of and disapproval of OHS compliance and risk perception by hospital top management and sanitary staff. As a result, these kinds of workplace hazard causes ought to be controlled by mild risk mitigation together with attentive observation and follow-up (Sup. Two: Table 1). The third type of risk identified occupational risk or hazards were tolerable risk within the hospitals that contributed 28% of the identified items. These type of occupational hzards included lack or poor practice of occupational health and safety service, substance abuse such as alcohol use, and chewing *Khat*. These type of hazard need tolerable risk mitigation with monitoring and follow up management (Sup. Two: Table 1).

Keep in mind that, the identification of potential risks at work has been prioritized for the risk mitigation (Fig. 4). In this study, RASM model was supposed to be met this risk mitigation by taking into account three dimensions of OHE sources such as hospitals, work environments, and SWs. Figure 4, at step 5, a safety factor (Safe = green) that is fulfilled when it should avoid unfavorable recognition for SWs, avoid not providing PPE, and avoid not providing a limited location for PPE (Fig. 4). Thus, RASM model by implement considering four measurement indicators [29] and five multi-modal strategies for sustainability [28]. The four indicators were proposed to evaluate implementation RASM model in the hospitals using process indicators [P], organizational indicators [O], individual indicators [I], and task and resource indicators [T]], adapted from [29], which are details (Sup. Two: Table 2). Then, it can be reported using standard, on improvement, proactive, reactive and naïve (none) criteria. Moreover, the overall sustainable of RASM model, five multi-modal strategies such as system change education and training, monitoring and feedback, communications and reminders, and safety climate and culture change are suggested, which was adapted from [30], details in supplementary (Sup. Two: Table 2). Figure 4 also provided a philosophy of tripartite structure that gives an equal voice to employees workers, employers and governments to ensure that the views of the social partners are closely reflected in labor standards and in shaping policies and programs [27].

## Strengths and limitation

### Strengths of the study

Despite the fact that it was a cross-sectional research, it has high and good strength, which helps to keep the paper’s quality. Among these are the scientific foundation and rationale for the investigation. Only by giving comprehensive and transparent information on all aspects of a cross-sectional study can the potential value of its findings and the risk of bias be appropriately assessed. The setting, locations, recruiting hours, and data collection were all assessed. The qualifying requirements were examined, as well as the sources and processes for participant selection were properly done.

### Limitations of the study

This study has certain limitations that should be highlighted. Because the data in this study were cross-sectional, a causal relationship could not be established. Future study should employ a prospective design to provide more robust proof of causality between linked variables and the development of hazards. There is little information available on the long-term consequences of workplace risks, as well as the effects of chemical and blood-borne pathogen exposure on worker health. Understanding the possible exposures and consequences connected with this employment can assist local, state, and federal governments in recognizing the need of emphasizing risks.

## Conclusion

According to the study’s findings, sanitary workers face a variety of OHE, including biological, chemical, ergonomics, physical, psychological, mechanical, and electrical hazards. As a result of these accumulated pressures, the proportion of sanitary personnel who self-reported occupational risks exposure was larger than three-fifths. Multiple logistic regression shows that sanitary workers without social recognition in hospitals, with an unpleasant attitude for PPE, with a history of sickness, little supervision, and dissatisfaction with the work environment were considerably more likely to increase the likelihood of occupational risks. As a result, hospitals, regional health bureaus, and the federal ministry of health and social affairs should take these predictions into account in order to mitigate via interventions such as OHS training and to provide a safe work environment for sanitary personnel.

### Supplementary Information

Supplementary Material 1.

Supplementary Material 2.

## Data Availability

The datasets used and analyzed during the current study available from the corresponding author.
