# Supplementary material for: Magnitude and associated factors of occupational hazard exposures among sanitary workers: Propose RASM model for risk mitigation for the public hospitals, eastern Ethiopia
Source: BMC Res Notes. 2024 Jun 20;17:172. doi: 10.1186/s13104-024-06828-2 (PMC11191316; doi:10.1186/s13104-024-06828-2)
Supplement: Supplementary file 1 — Supplementary Material 1. [file 13104_2024_6828_MOESM1_ESM.docx]

## **Study Design**

Hospital based cross-sectional study design was applied

## **Study Populations**

All hospitals sanitary workers working in all public hospitals found in Eastern Ethiopia were the sources of this study. While, all hospitals sanitary workers in selected governmental hospitals in eastern Ethiopia were study units

## **Inclusion and Exclusion Criteria**

All sanitary workers actively working such as cleaners and waste management workers actively working were included. All sanitary workers cleaning in patient wards and latrine /toilet, waste collectors or waste management workers, outsourced or contract as well as permanents having more than one-month work experience were assigned for the study. While, sanitary workers those were on annual, sick leave and maternal leave during study were excluded.

## **Sample Determination**

The sample size for prevalence of OHE among hospitals sanitary workers were estimated using single propor­tion formula:$N=\frac{z^{2}pq}{d^{2}}$, where: N is the required sample size, *Z* is the reliability coef­ficient at 95% confidence interval (1.96), *p* is the population proportion, *q* is equal to 1−*p*, and *d* is the acceptable error (0.05). As far as we concern, a study done in the country's neighbor, Egypt (1), where magnitude of occupational hazards among sanitary workers was 55%. As the result the sample size was n= $\frac{\left( 1.96 \right)^{2} \left( 0.45 \right)\left( 0.55 \right)}{\left( 0.05 \right)^{2}}$=380. Using design effect of 2.00, the sample size was 766. Adding with 5% contingency (38 individual), the final sample size was 798. This figure is approaching to all sanitary workers in eight hospitals (N=809). Thus, all units were recruited for the final study

## **Selection Procedures**

As illustrated above, eight public hospitals were selected at random from a total of fourteen public hospitals located throughout the regional states, with an equal probability allocated to each study location. Accordingly, a total of 234, 175, 82 and 318 SWs were eligible from HFCSH and JGJ (Harari regional state), BGH and CGH (Oromia regional state), DRF and SGH (Dire Dawa city administration) and from JUSHRH and KGH (Somali regional state), respectively (Figure 3).


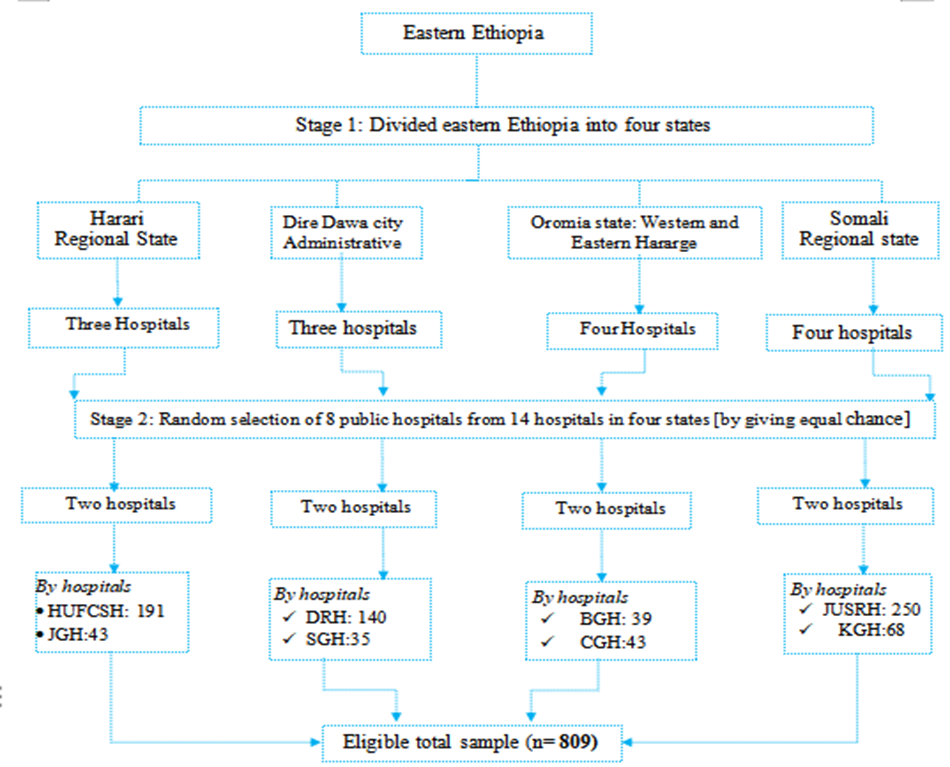


Figure 3 Schematic representative of sampling procedure of hospital SWs, eastern Ethiopia, 2023

**Keys:**

- *Asterisk* (*) stands for number of HSWs after data collection
- **HUCSH:** Haramaya University Hiwot Fana comprehensive specialized hospital’ **JGH:** Jugola general hospital, DRH: Dilchora Referral Hospital, **SGH**: Sabain general hospital; **JUSHRH**: Jigjiga University Sheik Hassen referral hospital, **KGH**: Karamara general hospital; **BGH**: Bisidimo general hospital, **CGH**: Chiro general hospital.

## **Study Variables**

The dependent variable was occupation hazard exposures , which could be happened as a result of unsafe working condition, acts due to poor practice of occupational health and safety service in the public hospital settings, adapted from (2, 3). While, the independent variables also associated factors or determinants, which are detailed as below. *Associated Factors questions*: A standard questions were adapted for associated factors of occupational hazard exposures(2, 3). These includes: *Individual factors*: knowledge and attitude towards workplace risks dichotomous questions that measured by YES/NO, and changed into good (16–20 scores); 2: Fair (10–15 scores); and 3: Poor (< 10 scores). Also attitude towards workplace risks questions measured by 1-5 likert scale: Strongly agree to Strongly disagree. Then, using media, it was classified as favored if score is higher than 3.0, neutral if score is equals to 3.0 and unfavored if score is less than 3.0 *Individual*/*Behavioral Factors*: four questions using Boolean logic either YES [1] or NO [0] were prepared to assess the sleep disorders, heavy alcohol consumption, chewing Khat and smoking cigarette. *Institutional Factors*: Questions were prepared Boolean Logic either YES [1] or NO [0] to assess regular supervision of OHS and training. For conducting supervision that can be categorized as no supervision, sometimes and daily supervision*. S*ocial recognition from the community or working settings could be poor [ negative] or good [positive], adapted from(4). *Conducive work environment satisfaction*~~:~~ is mainly refers to the safety, comfort, and harmony of the objective hospital environment in which they work, excluding salary and promotion factors. While unsafe work environment shows individual might be observed or perceived the workplace unsafe or hazardous workplace (5). *Workload:* A single item was asked and then enforced to answer (YES [1]/NO[0]) adopted from (6).

## **Data Collection methods**

***Pattern of the study:*** The pattern of the study was carried out into three types of shifts or job rotation for consecutive one month. Namely, first shift (Morning), second shift (Afternoon) and third shift (night). All hospital SWs with in the rotation of three shifts per week. The first shift starts at 7:00am and ends at 12:00am~~.~~ The second shift starts at 1:00pm and ends at 5:00pm. The third shift starts at 12:00pm to 6:00am. This group works on schedule for two consecutive days and then rests for two days~~]~~. By considering this, the questionnaires were administered (Morning: 9:00am-10:00am) for shift 1. For shift 2, the interview was done (Afternoon: 3:00pm-4:00pm]. The same procedure was done for shift 3 after 2 days.

***Data collectors:*** Eight data collectors participated in data collection. All of them have Master degree in Occupational health and safety and environmental health and public health.

***Data collection Tools:***

The research was conducted in mixed of quantitative and qualitative methods. The quantitative methods have their rational foundation in the positivist, while the qualitative methods have their rational foundation in Interpretivist (7, 8). Structured and standard closed questionnaires were used to collect quantitative data. Contents of the questionnaire include:

**Key Informant Interview (KII):**

Eight (8) Infection prevention and control focal persons were involved as KII in this study from eight hospitals. They selected purposely based on their expert and experience in the hospitals. The assumption was consisted in an in-depth and holistic fashion, through the collection of rich narrative materials using a flexible research assessment, adapted from (9). A structured and standard questions were prepared for the evaluation to elicit the details of response requires the respondents’ opinion on type of occupational hazards. Accordingly, thirty two (32) questions were prepared and all items were computed as *Severe* (S) X *Exposure* (E) X *Probability (P)=* (SEP) according to validated study (10). Where, *Severity(S)* is the impact of the events, categorized as slight effect ranged from 1 to 5 (Score: 1), minor severe with absence illness (2), moderate injuries requiring hospital admission (3), major injuries and illness in permanent (4) and death (5). *Exposure(E) is the frequency* of events ranged from 1 to 5, categorized as very rarely, year (Score=1) monthly (2), weekly (3) and daily (4), constantly, multiple times a day (5). *Probability(P) is the likelihood* of the events ranged from 1 to 5, categorized as chance to occurs is not expected through life of the work (Score=1), it indicates chance to conceivable is between 10-30 of working years (2), chance between 1-10 working years (3), chance within a month (4), and chance to occurs daily to weekly (5). The output of SEP all items were rated as highly high (If 90-100% scores rated as 5 ), high (if 80-89% scores rated as 4 ), medium (if 60-79 rated as 3 ) , low (if 50-59 score , rated as 2) and very low (If <50% score , it is rated as 1), adapted from (10).

***Propose health and safety risk Mitigation***

In this study, RASM model was proposed considering the following steps.

*Step 1: OHS hazards identification:* Eight (8) questions were prepared for SWs and thirty-two [32] questions were prepared for hospitals IPC experts. *Step 2: Risk Criteria determination:* The risk criteria determined by severity (S), exposure (E) and probability of the hazard to occur when exposed (P). *Step 3: Determine Risks Index:* It was determined by multiplying the parameters: R = (S x E x P), obtained from Curtis (11). *Step 4: Determine OHS actions for prioritized risk:* Step /Stage 1 to 4 also known as experts or professional stage. After risk prioritized, the mitigation will be based on the risk index mentioned above. *Step 5: Develop safety factors:* This stage works according to the priorities or gaps considering three dimension: Environmental, equipment and human factors obtained from Curtis (11). *Step 6:* It would be expected to implement multi-modal strategies (12) and the health and safety risk management indicators (13) and tripartite scenario (14) for the sustainability and monitoring of the risks. Stage 5 to 6 also known as managerial stage.

## **Data Quality**

The development of the questionnaires was guided by reviewed literature related to compliance with the OHS conditions and data collection methods. To ensure the quality of the data, standard and structure questionnaires, recruited profession data collectors, provide appropriate training and checked the reliability and validity of data (15, 16), conducted pretest study(Outside of study areas). In addition, the reliability of internal consistency of items for each objective was measured by Cronbach’s alpha value, where cut point was 0.70 < is “acceptable” (17). In addition, the correlation of item-test was presented according to Piedmont et al(18).

## **Data analysis**

The data was coded and exported into Epi data 3.1 before being exported to Stata 17. Descriptive statistics were used to characterize one independent or dependent variable. It was presented by mean, median, and standard deviations (continuous variables) as well as it was used for frequencies, proportions and percentages (categorical variables). In order to allow residual variables at the individual and hospital levels, the multilevel model (MLM) was employed. MLM multivariate logistic regression analysis was used determine the relationship between magnitude of occupational hazard exposures and independent variables. MLM model was performed with the following two criteria set at null model (model 0), adapted from (19). 1^st^ , the likelihood ratio of Chi^2^ -p-value should be < p-value of 0.05. 2^nd^, the value of Intraclass correlation coefficient (ICC) should be higher than 10%. Four models—the null model (model 0), the individual level variable (model 1), the hospital variables (model 2), and the combination of Models 1 and 2 (model 3)—were fitted to all outcomes. Then using likelihood ratio formula: -2 [log$\frac{L(\theta H0)}{L(\theta MLE)}$]= -2[Log$L(\theta H0)-logL(\theta MLE)]$, where, L=likelihood value, θ_H0_=null hypothesis estimate parameter, θ_MLE_= estimate parameter at data. Then it was selected the optimal model that has largest likelihood ratio at each model. The crude odds ratio (COR) and adjusted odds ratio (AOR) with a 95% confidence interval (95%CI) were presented at model 3. However, because quite a few of tables, table for models 1 and 2 were left out. The results of fixed effects for variables < 0.05 were reported as AOR at their 95% CI. Nested model with fixed effect and random effect was used to estimate regression coefficient (observable parameters) and ICC used to explore the unobservant variance among selected hospitals at 95%CI. The multicollinearity was also examined using the variance inflation factor (VIF), which measured how an independent variable's variance is inflated/correlated with the other independent variables, with a cut-off point of less than 10 (VIF).

**Reference**

1. Manal Fattah A, Salwa A, Wagida W, al e. Occupational Health Hazards among Sewage Workers. Zagazig Nursing Journal. 2016;12(2):204-18.

2. Olorunnishola O, Kidd-Taylor A, L. B, al e. Occupational injuries and illnesses in the solid waste industry: a call for action. New Solut. 2010;20(2):211-23.

3. Bogale D, kumie A, Tefera W, al. e. Assessment of occupational injuries among Addis Ababa city MSW collectors. BMC Public Health. 2014;14:150-69.

4. Bering I. The Meaning of Cleaning – on the subjective meaning of work”, ESREA conference New Research Themes on Learning and Work Life 19– 22 October, 2000; Seville2000.

5. Dolbier C, Webster J, McCalister K, Mallon M, Steinhardt M. Reliability and validity of a single-item measure of job satisfaction. Am J Health Promotion 2005;19:194-8.

6. Alamgir H, Shicheng Y. Epidemiology of occupational injury among cleaners in the healthcare sector. Occup Med (Lond). 2008;58(6):393-9.

7. Lin A. Bridging Positivist and Interpretivist Approaches to Qualitative Methods. Policy Studies Journal. 1998;26(1):162-80.

8. Ibrahim R. <https://wwwlinkedincom/pulse/combining-qualitative-and-quantitative-approaches-in-research/> [Internet]2014 December, 19th, 2022. [cited 2022].

9. Speziale S, Carpenter D. Qualitative Research in Nursing : Advancing the Humanistic Imperative Helen J. Streubert Speziale DRC, editor. Philadelphia: Lippincott Williams & Wilkins,PA Print.; 2003.

10. Wu ZJ, Xu B, Jiang H, Zheng M, Zhang M, Zhao WJ, et al. [Application of three risk assessment models in occupational health risk assessment of dimethylformamide]. Zhonghua Lao Dong Wei Sheng Zhi Ye Bing Za Zhi. 2016;34(8):576-80.

11. Curtis R. Risk Assessment & Safety Management (RASM): The Complete Risk Management Model for Outdoor Programs; OutdoorEd.com. 2015.

12. WHO WHO. Infection prevention and control assessment framework at the facility level. WHO; 2016.

13. Kaassis B, Badri A. Development of a Preliminary Model for Evaluating OHS Risk Management Maturity in Small and Medium-Sized Enterprises. MDPI_Safey 2018;4(5):01-20.

14. ILO ILO. International Labor Organization on tripartite govenrment , emplyers and employees responsibilites. Internet (Accessed 2 Sep.2023), available at <https://www.ilo.org/global/about-the-ilo/lang--en/index.htm#:~:text=The%20unique%20tripartite%20structure%>. 2023.

15. Polit D, Beck C. Generating and Assessing Evidence for Nursing Practice. 8th ed. Lippincott: Williams and Wilkins; 2008.

16. Sekaran U, Bougie R. Research Methods for Business. 5th ed. New Delhi:: John Wiley & Sons Ltd; 2010. 1-468 p.

17. George D, Mallery P. SPSS for Windows step by step: A simple guide and reference 11.0 update. 4th ed. Bacon A, editor. Boston2003.

18. Piedmont RL. Inter-item Correlations. In: Michalos AC, editor. Encyclopedia of Quality of Life and Well-Being Research;10.1007/978-94-007-0753-5_1493. Dordrecht: Springer Netherlands; 2014. p. 3303-4.

19. Koo T, Li MA. Guideline of Selecting and Reporting Intraclass Correlation Coefficients for Reliability Research. PMC4913118. EMEiJCMDPP, editor2016. 155-63. p.
