# Supplementary material for: Magnitude and associated factors of occupational hazard exposures among sanitary workers: Propose RASM model for risk mitigation for the public hospitals, eastern Ethiopia
Source: BMC Res Notes. 2024 Jun 20;17:172. doi: 10.1186/s13104-024-06828-2 (PMC11191316; doi:10.1186/s13104-024-06828-2)
Supplement: Supplementary file 2 — Supplementary Material 2. [file 13104_2024_6828_MOESM2_ESM.docx]

**Supplementary Two**

Sup-Table 1 Infection prevention and controls’ expert’s evaluation of occupational hazard priority among public hospitals in eastern Ethiopia, 2023

| **Type of Hazards** | **Sources** | **Risk calculation** | **Risk**  **Index** | **Type of risk** |
| --- | --- | --- | --- | --- |
| Musculoskeletal disorders due to bending, straightening, pushing, pulling, twisting clenching and reaching | E | RI: SEP+RIM | 385.13 | Significant Risk |
| Occupational injuries due to needle stick, broken glass, plastic and sharp materials | E | >> | 380.05 | Significant Risk |
| Occupational injuries due to improper medical waste management/MWM/ at sources | E | >> | 378.00 | Significant Risk |
| Poor Infection prevention and control member support are due to weak support of top management | I | >> | 366.38 | Significant Risk |
| Poor knowledge and un-favor of hospital’s top managers towards PPE compliance | I | >> | 360.81 | Significant Risk |
| Exposure to chemical detergents, solvents and hazardous materials due to lack of PPE | I | >> | 345.56 | Significant Risk |
| Exposure to ergonomically hazards like improper positioning and awkward posture | S | >> | 333.13 | Significant Risk |
| OHS problems due to inappropriate utilization of personal protective equipment | S | >> | 329.38 | Significant Risk |
| Exposure to physical hazards like insufficient light, noise, illustration | E/I | >> | 324.89 | Significant Risk |
| Job stress for sanitary workers due to unsatisfactory of work and environment | E | >> | 278.25 | Significant Risk |
| OHS problems due to insufficient personal protective equipment/PPE | I | >> | 269.88 | Significant Risk |
| OHS problems due to lack of occupational health and safety training for workers | I | >> | 267.50 | Significant Risk |
| OHS problems due to exposure to poor recognition for sanitary workers | E | >> | 249.94 | Significant Risk |
| Poor knowledge and un-favor of hospital’s sanitary workers towards PPE compliance | I | >> | 235.38 | Significant Risk |
| Occupational injuries due to poor transportation of medical waste management | E | >> | 219.63 | Significant Risk |
| Develop Occupational illness due to lack of post exposure prophylaxis | I | >> | 217.00 | Significant Risk |
| Exposure to biological hazards like HIV/AIDS, Hepatitis B and other pathogens | I | >> | 207.50 | Significant Risk |
| OHS problems due to poor practice of OHS and lack of its guideline | I | >> | 206.76 | Significant Risk |
| Occupational health and safety outcomes due to poor IPC member support | I | >> | 211.51 | Significant Risk |
| Poor knowledge and un-favor of hospital’s top managers towards OHS compliance | I | >> | 154.00 | Moderate Risk |
| Occupational injuries due to poor passageway for transportation of MW management | I | >> | 145.56 | Moderate Risk |
| OHS problems among sanitary workers due to lack of work experience in sanitation | I | >> | 112.00 | Moderate Risk |
| OHS problems due to personal protective equipment safety management/PPE | S/I | >> | 86.63 | Moderate Risk |
| OHS problems among sanitary workers due to education, salary and marital status | I | >> | 73.93 | Moderate Risk |
| Occupational injuries due to fall, slip, hit, caught equipment and materials | E | >> | 85.43 | Moderate Risk |
| Exposure to mechanical hazards like medical equipment and heavy material handling | E | >> | 72.21 | Moderate Risk |
| Poor knowledge and un-favor of hospital’s sanitary workers towards OHS compliance | S/E | >> | 74.13 | Moderate Risk |
| OHS problems due to unsafe behavior, alcohols consumptions and chewing chat | S | >> | 71.38 | Moderate Risk |
| Lack of safety awareness and conscious of workers on the job-related OHS issues | I | >> | 64.88 | Tolerance Risk |
| OHS problems due to irregular work schedules of workers (extended work hours) | I | >> | 55.96 | Tolerance Risk |
| Occupational exposure to electric hazards like ungrounded installations | I/E | >> | 38.00 | Tolerance Risk |
| Talking about bad things about sanitation and hygiene workers at hospital lead to them mental health | E | >> | 47.88 | Tolerance Risk |

**E**=Environment**, I**=Institutions/Hospitals, **S**=Sanitary workers, **RI**: Risk index, **S**: Severity, **E**: Exposure, **P**: Probability, **RIM**: Relative importance of management

Sup. Table 2. Infection prevention and control experts’ (Evaluators) profile in public hospitals, 2023

| S.no | Level of Education | Background education | Work experience in hospital |
| --- | --- | --- | --- |
| 1 | Master Degree | BSC OHS, MHP | 10 Years |
| 2 | Bachelor Degree | BSC EHS | 4 Years |
| 3 | Master Degree | BSC EHS, MHP | 8 Years |
| 4 | Master Degree | BSC EHS, Mcs | 7 Years |
| 5 | Master Degree | BSC HO, MPH | 7 Years |
| 6 | Bachelor Degree | BSC OHS | 16 Years |
| 7 | Bachelor Degree | BSC EHS | 5 Years |
| 8 | Bachelor Degree | BSC EHS | 3 Years |

Sup. Table 3 Source of occupational hazards in selected public hospitals in Eastern Ethiopia, 2023

| Source of hazard | Frequency | Percent | Cumulative |
| --- | --- | --- | --- |
| Institution/hospital (Inst.) | 16 | 50 | 84.38 |
| Work environment (Env.) | 9 | 28.13 | 28.13 |
| Sanitary workers (SWs) | 3 | 9.38 | 93.75 |
| Institution and Env | 2 | 6.25 | 34.38 |
| Sanitary workers and Env | 1 | 3.13 | 96.88 |
| Sanitary workers and Inst. | 1 | 3.13 | 100 |

**RASM model development and sustainability**

Sub-table 4 Proposed evaluation type for Risk assessment and safety management (RASM) any workplace

| Indicators and multimodal strategies | Evaluation type | Point | Points | Remarks |
| --- | --- | --- | --- | --- |
| **Four indicators [1]** |  |  | 50% |  |
| Process based indicators [P] | Standard of OHS | 12.5 |  |  |
|  | Improve OHS | 6.25 |  |  |
|  | Proactive OHS risk | 3.125 |  |  |
|  | Reactive OHS risk | 1.563 |  |  |
|  | Naïve to OHS | 0.781 |  |  |
| Organizational based indicators [O] | Standard of OHS | 12.5 |  |  |
|  | Improve OHS | 6.25 |  |  |
|  | Proactive OHS risk | 3.125 |  |  |
|  | Reactive OHS risk | 1.563 |  |  |
|  | Naïve to OHS | 0.781 |  |  |
| Individual based indicators [I] | Standard of OHS | 12.5 |  |  |
|  | Improve OHS | 6.25 |  |  |
|  | Proactive OHS risk | 3.125 |  |  |
|  | Reactive OHS risk | 1.563 |  |  |
|  | Naïve to OHS | 0.781 |  |  |
| Task and resource-based indicators [T] | Standard of OHS | 12.5 |  |  |
|  | Improve OHS | 6.25 |  |  |
|  | Proactive OHS risk | 3.125 |  |  |
|  | Reactive OHS risk | 1.563 |  |  |
|  | Naïve to OHS | 0.781 |  |  |
| **Five multimodal strategies [2]** |  |  | 50% |  |
| System change | % of changed tackles | 10 |  |  |
| Education and training | % of persons get | 10 |  |  |
| Monitoring and feedback | % of report | 10 |  |  |
| Communications and reminders | No of all sites | 10 |  |  |
| Safety climate and culture change | % of cultured safety | 10 |  |  |

Legend: Level >75: Good OHS compliance, 50-74: Medium OHS compliance, < 50 OHS compliance

[1] Kaassis B, Badri A. Development of a Preliminary Model for Evaluating OHS Risk Management Maturity in Small and Medium-Sized Enterprises. MDPI_Safey 2018;4(5):01-20.

[2] WHO/World Health Organization. Infection prevention and control assessment framework at the facility level. WHO; 2016.
